# Central Role of Glucocorticoid Receptors in Alzheimer’s Disease and Depression

**DOI:** 10.3389/fnins.2018.00739

**Published:** 2018-10-16

**Authors:** Geoffrey Canet, Nathalie Chevallier, Charleine Zussy, Catherine Desrumaux, Laurent Givalois

**Affiliations:** ^1^Molecular Mechanisms in Neurodegenerative Dementia Laboratory, INSERM, U1198, Team Environmental Impact in Alzheimer’s Disease and Related Disorders (EiAlz), Montpellier, France; ^2^University of Montpellier, Montpellier, France; ^3^EPHE, Paris, France

**Keywords:** Alzheimer’s disease, depression, risk factor, HPA axis, glucocorticoids, selective GR modulators

## Abstract

Alzheimer’s disease (AD) is the principal neurodegenerative pathology in the world displaying negative impacts on both the health and social ability of patients and inducing considerable economic costs. In the case of sporadic forms of AD (more than 95% of patients), even if mechanisms are unknown, some risk factors were identified. The principal risk is aging, but there is growing evidence that lifetime events like chronic stress or stress-related disorders may increase the probability to develop AD. This mini-review reinforces the rationale to consider major depressive disorder (MDD) as an important risk factor to develop AD and points the central role played by the hypothalamic-pituitary-adrenal (HPA) axis, glucocorticoids (GC) and their receptors (GR) in the etiology of MDD and AD. Several strategies directly targeting GR were tested to neutralize the HPA axis dysregulation and GC overproduction. Given the ubiquitous expression of GR, antagonists have many undesired side effects, limiting their therapeutic potential. However, a new class of molecules was developed, highly selective and acting as modulators. They present the advantage to selectively abrogate pathogenic GR-dependent processes, while retaining beneficial aspects of GR signaling. In fact, these “selective GR modulators” induce a receptor conformation that allows activation of only a subset of downstream signaling pathways, explaining their capacity to combine agonistic and antagonistic properties. Thus, targeting GR with selective modulators, alone or in association with current strategies, becomes particularly attractive and relevant to develop novel preventive and/or therapeutic strategies to tackle disorders associated with a dysregulation of the HPA axis.

## General Aspects

Alzheimer’s disease is the principal neurodegenerative pathology in the world. This pathology is characterized by a progressive impairment of cognitive functions associated with synaptic and neuronal loss, the presence in the brain of senile plaques and NFT. Plaques are composed of insoluble extracellular aggregates consisting principally of Aβ peptides, while NFT result from intracellular hyper- and abnormal phosphorylation of the microtubule-stabilizing protein Tau (Selkoe, 2001; Mattson, 2004).

**FIGURE 1:**
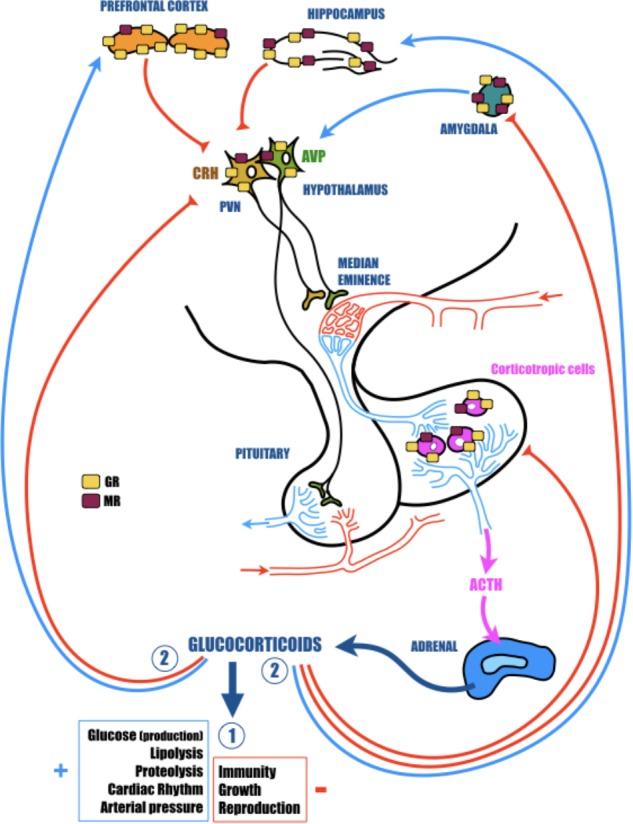
The hypothalamic-pituitary-adrenal (HPA) axis plays a vital role in adaptation of the organism to homeostatic challenge. The activation of the HPA axis by stress leads to rapid secretion of corticotropin releasing hormone (CRH) and arginine vasopressin (AVP) from the hypothalamic paraventricular nucleus (PVN). In turn, CRH and AVP activate the secretion of adrenocorticotropic hormone (ACTH) from the anterior pituitary, which finally stimulates the release of glucocorticoids (GC; cortisol in primates and corticosterone in rodents) from the adrenal glands. CRH is principally synthesized in the parvocellular portion of the PVN where it is co-localized with AVP. AVP acts in synergy with CRH and potentiates the ability of CRH to induce ACTH secretion. AVP is largely synthesized in the other portion of the PVN, the magnocellular region. Here, this hormone is preponderantly conveyed to the posterior pituitary, where it is stocked until release into the general circulation to participate in several actions particularly related to water control. GC readily cross the blood-brain barrier and bind to low-affinity glucocorticoid receptors (GR) and high-affinity mineralocorticoid receptors (MR). These nuclear receptors are indispensable for regular cellular activity and crucial for many central nervous system functions, including learning and memory. While MR are essentially localized in the hippocampus, GR are more ubiquitous and are particularly found in the hypothalamus and in the pituitary, but also in several structures of the limbic system (prefrontal cortex, hippocampus, and amygdala), which are highly involved in cognitive and psychological functions. All of these cerebral regions are important components of the neural circuitry mediating HPA axis activity. Indeed, while hippocampus and prefrontal cortex exert a tonic inhibition, amygdala stimulates hypothalamic neurons. In fact, though structural plasticity in hippocampus and prefrontal cortex might mediate cognitive impairment caused by severe stress, changes in amygdala are more likely to contribute to the affective aspect of stress disorders. In response to homeostatic challenge, GC stimulate in a first time (Circle 1) all physiological functions necessary to induce an adapted response to the stress (cardiovascular and metabolic) and inhibit all functions non-immediately necessary (immunity, reproduction, and growth). In a second time (Circle 2), to avoid a runaway of the HPA axis, GC exert a negative feedback at all levels of the axis (limbic, hypothalamic, and pituitary).

There are several forms of AD. Familial forms with known mutations of specific genes, representing less than 5% of AD cases, and sporadic forms representing more than 95% of patients, with unknown mechanisms, but identified risk factors. The principal risk factor for sporadic AD is aging. The risk doubles every 5 years after age 65, and prevalence reaches 50% over the age of 85. There is also growing evidence that lifetime events like chronic stress or stress-related disorders may increase the probability to develop AD (Heininger, 2000; Blennow et al., 2006; Querfurth and Laferla, 2010). This view is particularly supported by the fact that in AD patients, psychological symptoms and cognitive deficits are associated with an early dysregulation of the HPA axis, which is highly involved in stress responses (de Kloet et al., 2005; **Figure 1**). In AD, HPA axis dysregulation is associated with elevated levels of GC (cortisol in human and corticosterone in rodent) in plasma and cerebrospinal fluid (Hartmann et al., 1997; Swanwick et al., 1998; Csernansky et al., 2006; Hoogendijk et al., 2006).

Glucorticoids are steroid hormones that freely cross the blood–brain barrier and bind to high-affinity (Kd = 0.5 nM) MR and low-affinity (Kd = 5 nM) GR (Reul and de Kloet, 1985). Globally, MR are necessary for regular cellular activity, but are also the receptors for aldosterone (involved in specific cells to the enzymatic degradation of GC). By contrast, GR are involved in stress responses, exert a negative feedback on the HPA axis activity and are essential for many CNS functions, including learning and memory (Roozendaal, 2000). While MR are essentially localized in the hippocampus, GR are more ubiquitous. They are particularly found in several structures of the limbic system (prefrontal cortex, hippocampus, and amygdala), which are especially involved in psychological and cognitive functions, but also are important elements of the neural circuitry mediating HPA axis activity (Jankord and Herman, 2008; **Figure 1**). Therefore, while structural plasticity in the hippocampus and prefrontal cortex may mediate cognitive impairment induced by severe stress, modifications in amygdala are more likely to contribute to the affective aspect of stress disorders (Vyas et al., 2004). Furthermore, because GC act synergistically with excitatory amino acids (like glutamate), disruptions of the HPA axis, GC overexposure or a modification of GR functioning could be extremely toxic, particularly in the limbic structures (hippocampus, prefrontal cortex, or amygdala) and thus contribute to the cognitive decline associated with stress-related disorders (McEwen, 2008). For instance, it was established a long time ago that chronic stress and subsequent GC over-secretion severely impact the structure, function and plasticity of synapses in the hippocampus. Repeated stress causes atrophy of dendrites in the CA3 region, and both acute and chronic stress suppress neurogenesis in dentate gyrus neurons (McEwen and Sapolsky, 1995; Galea et al., 1997; McEwen, 1999; Vyas et al., 2002). This loss of synaptic plasticity is in the heart of AD (Selkoe, 2002), and could be in part responsible for the cognitive decline observed in patients, and thus making a link between stress, stress-related disorders, GC, and AD.

As previously established by Ownby et al. (2006) and recently reviewed by Ishijima et al. (2018), epidemiological studies demonstrated that MDD (the stress-related disorder by excellence) may be considered as an important risk factor to develop AD (Ownby et al., 2006; Ishijima et al., 2018). Early life MDD (more than 25 years before the diagnosis of dementia) is correlated with a belated development of AD (Robert et al., 2003), and has systematically been associated with a more than twofold increase in dementia risk (Byers and Yaffe, 2011). In fact, the risk to develop AD increases with every new affective episode associated to mood disorders and especially, the level of dementia tended to increase by 13% with every episode of MDD (Kessing and Andersen, 2004). Additionally, numerous proofs suggest that late-life MDD also increases the risk to develop dementia (Baldwin et al., 2006; Thomas and O’Brien, 2008). It appears that MDD accelerates age-related cognitive decline (Gualtieri and Johnson, 2008) and promotes the conversion of mild cognitive impairments into AD (Modrego and Ferrández, 2004; Houde et al., 2008). Finally, MDD may occur in 30–40% of the AD patients (Assal and Cummings, 2002; Starkstein et al., 2005) and affects the clinical evolution of AD (Shim and Yang, 2006). Senile plaques and NFT are more marked in the hippocampus of AD patients with comorbid MDD as compared with AD patients without depression (Rapp et al., 2008).

However, even though mechanisms of the switch from MDD to AD remain unclear, some findings suggest that one of the links between these two disorders could be a dysregulation of the HPA axis activity, associated with impaired GC signaling (Ownby et al., 2006; Caraci et al., 2010; Notarianni, 2013; Givalois, 2014; Herbert and Lucassen, 2016). Thus in the present review, we will examine this evidence, focusing on the HPA axis dysregulation in AD and MDD, and on the new molecules targeting selectively GR for the treatment of both MDD and AD.

## HPA Axis Dysregulation

The links between AD, HPA axis, stress and GC come from observations in humans, but also from different animal models. In humans, there is considerable evidence involving HPA axis dysfunction in AD patients. This dysregulation is reflected not only by elevated levels of circulating cortisol, but also by the failure to show cortisol suppression following a DEX challenge, suggesting the inability for the HPA axis to maintain homeostasis (Martignoni et al., 1990; Hartmann et al., 1997; Weiner et al., 1997; Swanwick et al., 1998; Csernansky et al., 2006; Elgh et al., 2006; Hoogendijk et al., 2006; Popp et al., 2009). Chronic stress, such as mourning or sleep deprivation, in addition to cause memory impairments, increases the susceptibility to develop AD (Mejía et al., 2003; Wilson et al., 2005, 2006). In AD patients treated with prednisone (a synthetic GC used for its anti-inflammatory properties), behavioral decline was increased when compared with the placebo-treated patients (Aisen et al., 2000). Besides, de Quervain et al. (2004) evidenced a haplotype in the gene of an enzyme involved in the activation of GC (11β-hydroxysteroid dehydrogenase – 11β-HSD) that increases by six the risk to develop AD (de Quervain et al., 2004).

In different Tg animal models of AD, chronic stress improved plaque pathology, accelerated the inception of cognitive deficits, triggered APP misprocessing, reduced Aβ clearance, increased Aβ levels, stimulated Tau hyperphosphorylation and its neuronal accumulation (Dong et al., 2004; Green et al., 2006; Jeong et al., 2006; Huang et al., 2011; Rothman et al., 2012). The presence of a GRE in the promoter regions of the APP and BACE1 genes (Lahiri, 2004; Sambamurti et al., 2004) may explain the impact of chronic stress and the role of GC in APP misprocessing and induction of the Aβ pathway. Regarding Tau, some data suggest that the modification of Tau system could be an indirect consequence of chronic stress, due to stress-induced Aβ increase (Tomidokoro et al., 2001; Tu et al., 2014). However it seems that chronic stress, or GC-excess could directly impact Tau phosphorylation (Yan et al., 2010), mainly through the over-activation of GSK-3β and Cdk5 enzymes (Papadopoulou et al., 2015; Dey et al., 2017; Yi et al., 2017). In addition, another factor linking chronic stress and Tau has also been identified, the corticotropin-releasing factor receptor (CRF1), since this receptor appears to be directly involved in the progression of Tau pathology (Rissman et al., 2007; Caroll et al., 2011).

In an acute model of AD, injection of an oligomeric solution of an Aβ fragment (oAβ_25-35_) in cerebral ventricles (icv) induces a wide pattern of central modifications reminiscent of the human pathophysiology (Zussy et al., 2011, 2013; Pineau et al., 2016). This Aβ fragment is found early in AD patients and originates from proteolysis of parent amyloid proteins (Kaneko et al., 2001; Kubo et al., 2002; Gruden et al., 2007). It also induces a strong and long-lasting activation of the HPA axis, which is associated with a modification of the expression and functioning of GR (Brureau et al., 2013; Pineau et al., 2016).

Interestingly, this deregulation of the HPA axis associated with AD, is also the most prevalent and well-documented neuroendocrine abnormality in stress-related disorders and especially in MDD (Holsboer and Barden, 1996). This pathology appears like a prodromal stage and an important element of AD, but could also be a trigger for developing AD (Herbert and Lucassen, 2016; Ishijima et al., 2018). In fact, in addition to be a risk factor in AD (Heininger, 2000; Blennow et al., 2006; Querfurth and Laferla, 2010), chronic exposure to stress and stressful life events also seem to lead to the development of MDD (Pariante, 2003; Charney and Manji, 2004; Czéh and Lucassen, 2007; Pittenger and Duman, 2008; Herbert and Lucassen, 2016). Taken together these data demonstrate a central role of HPA axis dysregulation and high levels of GC both in MDD and AD etiology and suggest the possibility that GR might be suitable targets both for antidepressant and antidementia drugs.

## GR, a Potential Therapeutic Target

**FIGURE 2:**
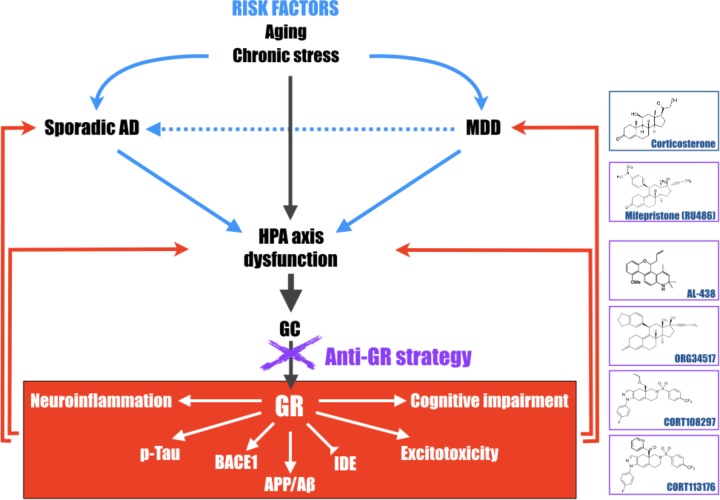
Schematic representation of the central role of the hypothalamic-pituitary-adrenal (HPA) axis dysfunction, GC and GR in the development of major depressive disorder (MDD) and Alzheimer’s disease (AD). Long-term exposure to stress or stress-related disorders (like MDD) contributes to cognitive impairment, Aβ accumulation, Tau hyperphosphorylation, excitotoxicity, and neuroinflammation processes, leading to development of AD. This schematic representation highlights that pathologies associated with a dysregulation of the HPA axis must be considered, with aging, as important risk factors to develop AD and evidence that therapies aiming at reducing high GC levels upstream, become particularly attractive and relevant in the treatment of the elderly, MDD or early AD patients. Chemical structures of molecules described in this review; corticosterone, mifepristone, AL-438 (Coghlan et al., 2003), ORG34517 (Bachmann et al., 2003), CORT108297, and CORT113176 (Pineau et al., 2016).

Based on the above-mentioned observations in humans and in animal models, suggesting a deregulation of GR functioning, several strategies targeting directly GR were tested in AD and MDD, and seem to have an important therapeutic potential (Bachmann et al., 2003; Caraci et al., 2010; **Figure 2**). However, given the ubiquitous expression of these receptors (Sapolsky et al., 2000), antagonists could have many undesired side effects and should be used with caution.

In MDD, but also in psychotic depression and bipolar disorder, preclinical and clinical studies were realized with the prototypical GR non-selective antagonist mifepristone (RU486) (**Figure 2**) and showed that this molecule seemed to be effective in inducing a rapid improvement of psychotic and depressive symptoms, while being well tolerated by patients (Belanoff et al., 2001, 2002b; Young et al., 2004; Oomen et al., 2007; Schatzberg and Lindley, 2008; Blasey et al., 2009; Wulsin et al., 2010; Howland, 2013; Block et al., 2017).

In AD, first studies with mifepristone provided very hopeful results. A chronic treatment in 3 × Tg-AD mice reversed cognitive deficits, clearly reduced Aβ levels, as well as phosphorylation and accumulation of Tau (Baglietto-Vargas et al., 2013). In Tg2576 mice, mifepristone rescued early episodic memory and synaptic plasticity deficits (Lanté et al., 2015). In the oAβ_25-35_ model, mifepristone restored basal circulating CORT levels, reversed synaptic deficits and apoptosis in the hippocampus. However, this non-selective antagonist only partially reversed cognitive and Aβ clearance deficits, hippocampal APP misprocessing, and neuroinflammatory processes, suggesting limits in its efficacy (Pineau et al., 2016). This limitation was also observed in humans since mifepristone, even if it slows the progression of cognitive decline in AD patients (Belanoff et al., 2002a), increases morning levels of blood GC (Pomara et al., 2006), suggesting potential side effects and thus a limited therapeutic usefulness.

Thus, several potent and selective GR ligand series were recently developed and seem to have an interesting therapeutic potential. The first non-steroidal selective GR molecules come from anti-inflammatory studies. Indeed, GC are generally prescribed in inflammatory diseases, however, chronic treatment with steroids leads to various undesired effects, most likely due to the wild range of genes targeted by GR and not directly involved in inflammatory processes. The objective of Abbott Laboratories (Abbott Park, IL, United States) was to create a series of molecules capable to have anti-inflammatory properties without deleterious side effects (Coghlan et al., 2003). The Abbott-Ligand 438 (AL-438) (**Figure 2**) was obtained by modifying a synthetic progestin scaffold resulting in the discovery of a series of high affinity, selective ligands for GR (Elmore et al., 2001). In comparison with prednisone, the steroidal anti-inflammatory molecule of reference, AL-438 shares the same affinity for MR as well as high affinity for GR as prednisone. Even if, in MR-dependent reporter gene assays, AL-438 showed low antagonist properties, whereas prednisone is a full agonist at nanomolar concentrations. *In vivo*, AL-438 preserved full anti-inflammatory efficacy and potency equivalent to steroids while side effects were especially reduced (Coghlan et al., 2003). Unfortunately, this compound and its derivatives were never tested, to our knowledge, in AD or MDD studies.

More recently, a new molecule was tested in an experimental model of ethanol dependence showing HPA axis and GR impairments (Reynolds et al., 2015). The ORG 34517 (**Figure 2**) is a 11,21-Bisphenyl-19-norpregnane steroid originally discovered by Organon (Oss, Netherlands). This compound, highly selective for GR (Peeters et al., 2008), has insignificant affinity for human PR, since it possesses a nearly 500-fold greater affinity for human GR (Gebhard et al., 1994). In addition, ORG 34517 is unable to occupy MR after an acute subcutaneous injection (Bachmann et al., 2003). At this time, this selective GR antagonist was envisaged as a promising therapeutic alternative in MDD (Bachmann et al., 2003). However, to our knowledge, this compound was never tested in MDD or in AD.

Another series of selective non-steroidal molecules (1H-pyrazolo[3,4-g]hexahydro-isoquinoline sulfonamides) come from Corcept Therapeutics (Menlo Park, CA, United States). These GR ligands and in particular CORT108297 and CORT113176 (**Figure 2**) present excellent affinity only for GR, and none for the other nuclear hormone receptors PR, AR, MR, and ER (Clark et al., 2008; Beaudry et al., 2014; Hunt et al., 2015; Pineau et al., 2016). Several cell-based assays were developed to assess their functionality. DEX, the GR agonist of reference, increases the activity and expression of TAT in liver cells. In HepG2 human cells or in human hepatocytes, both CORT108297 and CORT113176 act as full antagonists since they are able to prevent the DEX-induced increase in TAT activity and to induce non-measurable agonist activity in the absence of DEX. However, when tested in a similar assay in rat hepatocytes, both molecules display incomplete antagonism and partial agonist activity (Beaudry et al., 2014; Pineau et al., 2016). An additional cell-based functional assay was developed in the A549 cell line to investigate the effect of these two compounds on IL-1β-induced IL-6 production. Both ligands demonstrated partial agonist effects, and also acted as partial antagonists when tested in presence of DEX (Pineau et al., 2016). Thus, their particular modulator properties make this family of molecules really interesting in AD or MDD. In fact, these ligands have the capacity to more selectively abrogate pathogenic GR-dependent progressions in the brain, while retaining positive aspects of GR signaling. Onno Meijer’s team showed that CORT108297 clear antagonist effects on the brain were accompanied by a lack of negative-feedback inhibition of the HPA axis, which suggests “the possibility of antagonizing a number of GR effects without affecting systemic basal GC levels” (Zalachoras et al., 2013). In fact, it appears that this family of molecules acts as “selective GR modulators” rather than pure antagonists. They induce a receptor conformation that permits activation of only a subset of downstream signaling pathways, explaining their capacity to combine agonistic and antagonistic properties (Zalachoras et al., 2013; Meijer et al., 2018).

In a 3 × Tg mice model of AD, CORT108297 reduces APP-C-terminal fragment (C83) and Tau hyperphosphorylation via reductions in p25 levels (Baglietto-Vargas et al., 2013). Furthermore, in the acute model, we recently showed that treatments with CORT108297 and CORT113176 reverse the hippocampal amyloidogenic pathway induced by the icv injection of oAβ_25-35_ through the inhibition of the principal enzyme involved in Aβ synthesis (BACE1) and the increase of one enzyme mainly involved in the elimination of Aβ (IDE). In addition, selective GR modulators reestablish hippocampal levels of synaptic markers, reverse hippocampal apoptotic processes and neuroinflammation, re-establish basal plasma levels of GC and *in fine* cognitive functions (Pineau et al., 2016).

In rodents, CORT108297 treatment decreases immobility in the FST suggesting potential antidepressant properties (Solomon et al., 2014). By contrast, treatment with another member of this family, CORT118335, which is a GR modulator but also a MR antagonist, did not affect immobility in the FST (Nguyen et al., 2018), suggesting a differential specificity and efficacy of each molecule.

Thus, the difference of efficacy between all of these compounds could be due to the difference of selectivity and affinity for GR (Coghlan et al., 2003; Clark et al., 2008; Peeters et al., 2008; Beaudry et al., 2014; Hunt et al., 2015; Pineau et al., 2016), but also to the intrinsic properties of GR and their ability to differentially recruit nuclear receptor coregulators after ligands binding (Coghlan et al., 2003; Zalachoras et al., 2013; Atucha et al., 2015; Meijer et al., 2018). These coregulators are transcriptionally active proteins, which mediate the transcriptional properties of nuclear receptors. They have tissue-, ligand-, and cell-specific expression patterns, and display gene- and receptor-specific interactions (Meijer et al., 2000; Lachize et al., 2009; Zalachoras et al., 2013; Meijer et al., 2018). Recently, Onno Meijer’s team, established that each GR compound induced a specific profile of interaction with these coregulators. They suggested, as previously envisaged by Coghlan et al. (2003) that these specific profiles could explain the difference of functionality and efficacy of these particular GR ligands and their capacity to combine antagonistic and agonistic properties (Atucha et al., 2015; Meijer et al., 2018). Accordingly, as recently suggested by Meijer et al. (2018), a better knowledge of the specific molecular interaction profiles of each GR compound, combined with the regional distribution of each coregulator in the brain, could assist in dissecting the molecular signaling pathways underlying pathologies associated with high levels of GC. This strategy will participate to create new avenues of investigation on GC and GR, and to exploit these avenues to develop novel preventive and/or therapeutic strategies to tackle disorders (neurodegenerative or not), associated with a dysregulation of the HPA axis.

GR activity can also be indirectly modulated by side regulations which could be additional potential targets. It opens the door to multiple approaches to target the GR pathway. Recently, it was demonstrated that inhibiting the adenosine A2A receptor, which is upregulated in the forebrain of AD patients, reverses memory deficits through HPA axis feedback and corticosterone circadian levels reestablishment (Batalha et al., 2013). Authors also evidenced that A2A receptor is a major regulator of GR function since its inhibition reduces GR hippocampal levels, and acts on GR nuclear translocation and GR-dependent transcriptional regulation (Batalha et al., 2016). Interestingly, some studies showed an anti-depressive effect of A2A receptor antagonists in MDD models (López-Cruz et al., 2018; Padilla et al., 2018). A2A receptor is an example among others. Indeed, annexin A1 is a GC-induced molecule that is known to replicate many of the described anti-inflammatory effects of GC (Yang et al., 2013). Even if there is no study about the role of annexin A1 in MDD, emerging evidence suggest a role of this protein in the clearance and the degradation of Aβ peptides, and in the neuroprotective role of microglia (McArthur et al., 2010; Ries et al., 2016).

## Conclusion

All these findings in favor of the “GC theory” reinforce the hypothesis that long-term exposure to stress or stress-related disorders (like MDD or Cushing’s syndrome for instance), contributes to cognitive impairment, Aβ accumulation, Tau hyperphosphorylation, excitotoxicity, and neuroinflammation processes, leading to later development of AD. They also evidence that pathologies associated with a dysregulation of the HPA axis must be considered as important risk factors for AD (**Figure 2**). Therefore, therapies aiming at reducing high GC levels upstream, in the elderly or in early AD patients, could be envisaged. This review also evidences that modulator molecules targeting selectively GR could abrogate pathogenic GR-dependent processes induced by a dysregulation of the HPA axis and retain beneficial and primordial aspects of GR signaling. Thus, this class of compounds, alone or in association with current treatments against MDD (anti-depressant compounds) or AD (anti-NMDA and anti-cholinesterase molecules), becomes particularly attractive and relevant candidates in the treatment of stress-related disorders or neurodegenerative diseases, and particularly AD.

## Author Contributions

GC, NC, CZ, CD, and LG equally contributed to the definition of the scope and to writing of the manuscript.

## Conflict of Interest Statement

The authors declare that the research was conducted in the absence of any commercial or financial relationships that could be construed as a potential conflict of interest.

## Abbreviations

Aβ: amyloid-β peptide
AD: Alzheimer’s disease
APP: amyloid precursor protein
AR: androgen receptors
BACE1: β-APP cleaving enzyme type 1
Cdk5: cyclin dependent kinase 5
CNS: central nervous system
DEX: dexamethasone
ER: estrogen receptors
FST: forced swim test
GC: glucocorticoids
GR: glucocorticoid receptors
GRE: glucocorticoid response element
GSK-3β: glycogen synthase kinase 3β
HPA axis: hypothalamic-pituitary-adrenal axis
Icv: intracerebroventricular
IDE: insulin-degrading enzyme
MDD: major depressive disorder
MR: mineralocorticoid receptors
NFT: neurofibrillary tangles
oAβ_25-35_: oligomers of Aβ fragment [25-35]
PR: progesterone receptors
TAT: tyrosine aminotransferase activity assay
Tg: transgenic
